# Supplementary material for: Pathways to diagnosis of a second primary cancer: protocol for a mixed-methods systematic review
Source: BMJ Open. 2017 Dec 21;7(12):e017929. doi: 10.1136/bmjopen-2017-017929 (PMC5988126; doi:10.1136/bmjopen-2017-017929)
Supplement: Author's manuscript [file bmjopen-2017-017929.draft_revisions.pdf]

BMJ Open is committed to open peer review. As part of this commitment we make the peer review history of every article we publish publicly available.

When an article is published we post the peer reviewers' comments and the authors' responses online. We also post the versions of the paper that were used during peer review. These are the versions that the peer review comments apply to.

The versions of the paper that follow are the versions that were submitted during the peer review process. They are not the versions of record or the final published versions. They should not be cited or distributed as the published version of this manuscript.

BMJ Open is an open access journal and the full, final, typeset and author-corrected version of record of the manuscript is available on our site with no access controls, subscription charges or pay-per-view fees (http://bmjopen.bmj.com).

If you have any questions on BMJ Open's open peer review process please email editorial.bmjopen@bmj.com

BMJ Open

Pathways to diagnosis of a second primary cancer: protocol for a mixed methods systematic review

Journal:	*BMJ Open*
Manuscript ID	bmjopen-2017-017929
Article Type:	Protocol
Date Submitted by the Author:	25-May-2017
Complete List of Authors:	Kanguru, Lovney; University of Edinburgh, Usher Institute of Population Heath Sciences and Informatics<br>Bikker, Annemieke; University of Edinburgh, Usher Institute of Population Heath Sciences and Informatics<br>Cavers, Debbie; University of Edinburgh, Usher Institute of Population Heath Sciences and Informatics<br>Barnett, Karen; University of Edinburgh, Usher Institute of Population Heath Sciences and Informatics<br>Brewster, David ; Scottish Cancer Registry<br>Weller, David; University of Edinburgh, Usher Institute of Population Heath Sciences and Informatics<br>Campbell, Christine; University of Edinburgh, Usher Institute of Population Heath Sciences and Informatics
**Primary Subject Heading**:	Public health
Secondary Subject Heading:	Public health, Oncology, Health services research
Keywords:	Second primary cancer, diagnostic pathway, routes to diagnosis, diagnostic interval, patient and health-care provider experiences, heath-care provider challenges

SCHOLARONE™
Manuscripts

Title

Pathways to diagnosis of a second primary cancer: protocol for a mixed methods systematic review

Authors

Lovney Kanguru[1*], Annemieke Bikker[1], Debbie Cavers[1], Karen Barnett[1], David H Brewster[2], David Weller[1], Christine Campbell[1]

Affiliation

[1]Usher Institute of Population Heath Sciences and Informatics, University of Edinburgh, EH8 9AG

[2]Scottish Cancer Registry, Information Services Division, NHS National Services Scotland, Edinburgh EH12 9EB

***Corresponding Author**

Lovney.Kanguru@ed.ac.uk

For peer review only

Abstract

Introduction: As cancer survivors continue to live longer, the incidence of second primary cancers (SPCs) will also rise. Relatively little is understood about the diagnostic pathway for SPCs, how people appraise, interpret symptoms and seek help for a second different cancer, and the experiences (including challenges) of health-care providers relating to SPCs. This study aims to systematically appraise and synthesise the literature on the pathways to diagnosis of a SPC and the associated patient and health-care provider experiences.

Methods: The approach taken includes systematic searches of published and unpublished literature without any date or language restrictions. Medline, Embase, CAB Abstracts, Medline-in-process and non-indexed citations, PsycInfo, Epub Ahead of Print in-process & other non-indexed citations Ovid Medline Daily, CINAHL, ASSIA, Sociological Abstracts, Web of Science, PROSPERO and grey literature will be searched to identify observational, systematic reviews, mixed methods and qualitative studies of interest. Titles, abstracts and full texts will be screened against the inclusion - exclusion criteria by at least two reviewers independently. Relevant outcomes of interest and study and population characteristics will be extracted. Synthesis will be used guided by the pathways to treatment model, and the Olesen model of time intervals.

Ethics and dissemination: Ethical approval is not required. This systematic review will provide a deeper understanding of the complex and heterogeneous diagnostic pathways of SPCs, while identifying common themes across the diagnostic interval, routes to diagnosis and patient and health-care provider experiences. These findings will help provide a nuanced picture of the diagnostic pathway for SPCs that may inform policy and consistent practice in particular approaches to early diagnosis for a SPC including the timing and reasons behind the decision by the patient to seek care, challenges faced by health-care providers, and in the development of future interventions to reduce the delay in patient time-to-presentation.

Registration: PROSPERO CRD42016051692

For peer review only

Strengths and limitations of this study

- This is the first systematic review to appraise and synthesise the literature on the pathways to diagnosis of a SPC and the associated patient and health-care provider experiences
- This study may help provide a nuanced picture of the diagnostic pathway for SPCs that may inform policy and consistent practice in particular approaches to early diagnosis for a SPC including the timing and reasons behind the decision by the patient to seek care, challenges faced by health-care providers, and in the development of future interventions
- The review is not restricted to any time period or specific languages
- We anticipate that the available data from qualitative studies may be limited (as informed by an initial scoping exercise) and as such, meta-ethnography synthesis may not be possible to do
- It is unlikely that a meta-analysis will be applicable for the quantitative studies included because the scope of this review does not cover survival outcomes along the diagnostic pathway for SPCs

For peer review only

Introduction

The number of people surviving cancer is steadily increasing [1]. As cancer survivors continue to live longer, in common with the wider population, the incidence of second primary cancers (SPCs) is also expected to grow [2]. A second primary cancer has been defined as a new primary cancer that occurs in a person who has had cancer in the past [3]. Evidence suggests that the incidence of SPCs among cancer survivors ranges between 1% and 17% depending on the index cancer site [4 -13], with SPCs estimated to account for as much as 16-18% of total cancer incidence [14-16] in western countries.

Fear of a cancer recurring and the possibility of getting a new primary cancer or multiple primaries are often a source of worry and psychosocial distress among cancer survivors [17-19]. These cancer-related health worries have been linked to higher levels of mental illness particularly anxiety and depression in long-term survivors [17]. Furthermore, the fear of, and past experiences of cancer have been shown to affect patients' help seeking behaviour [20 -23]. Symptoms of a SPC may be missed if patients are not aware of their significance [20]. This could be because of patients focusing on the symptoms of the original cancer only, or patients' perceptions being influenced by the presence of co-morbid conditions [24, 25, 26].

Early detection of cancers is an important element in improving cancer outcomes, as the stage of disease at diagnosis is linked to survival for many cancers [20, 25]. While patients' ability to recognise possible warning signs and taking prompt action is a critical early step, evidence suggests that the patient diagnostic pathway is often not straightforward [27 - 31]. For example, the diagnostic interval may vary depending on the route to diagnosis, which has potential implications for the stage at diagnosis. The Pathways to Treatment Model [24] provides a theoretical framework for the wide range of factors that influence the pathway to diagnosis, not only those relating to the patient, but also those linked to the health-care provider, the health system and the cancer itself [Figure 1]. There are also key time-points in diagnostic journeys (Olesen et al [32]; Weller et al [33]) as illustrated in Figure 2.

For peer review only - http://bmjopen.bmj.com/site/about/guidelines.xhtml

Both models emphasise the often-complex journeys that patients go through from the onset of first symptoms to the start of treatment.

A greater understanding of patient pathways to diagnosis (including diagnostic routes) for a SPC is vital due to the growing incidence, yet comparatively little is known about these pathways. Mapping the evidence on pathways to diagnosis for a SPC and the associated patient and health-care providers' experiences will be an important starting point that will provide a nuanced picture of the diagnostic pathway for SPCs. These insights may help to inform policy and consistent practice in approaches to early diagnosis for a SPC, including the timing and the decision by the patient to seek care, and in the development of future interventions to reduce the delay in patient time-to-presentation.

This review aims to systematically appraise and synthesise the literature on pathways to diagnosis of a SPC and associated patient and health-care provider experiences. Our objectives are:

1. To summarise the literature describing pathways to diagnosis of a SPC, including any descriptions of patient and diagnostic intervals, and routes to diagnosis.
2. To summarise the literature describing how patients appraise symptoms and engage with health care in the pathway to diagnosis of a SPC
3. To explore the available evidence on health-care providers' perspectives around the pathways to diagnosis of a SPC and the challenges they face

For peer review only

METHODS

The PRISMA-P statement [34] has guided the development and reporting of this systematic review protocol.

Inclusion and exclusion criteria

Table 2 summarises the inclusion – exclusion criteria. This review will consider studies that report on any stage of the pathways to diagnosis for a SPC, including routes to diagnosis (e.g. during routine follow up or surveillance relating to the first cancer, through engagement with screening, symptomatic presentation in general practice or through emergency presentation). We will also consider studies that report on patient and diagnostic intervals such as symptom appraisal, help seeking behaviour, diagnostic and pre-treatment intervals [24, 28, 33]. We will follow the definitions of appraisal, help-seeking, diagnostic and pre-treatment intervals as defined in the Aarhus Statement [33]. Although these definitions are important in this review, we will not exclude studies because of lack of, or poor definitions only.

Studies that report on how patients appraise and interpret symptoms, engage with healthcare in the diagnosis of a SPC, and on routes to diagnosis, will also be included. Studies for inclusion will be limited to those that examine patients with SPCs only and where patients are 18 years and above at the time of first primary cancer diagnosis. We recognise that SPCs in adults following a childhood or adolescent primary cancer is an important area, and there is substantial work that has been done on late effects of childhood cancers that includes SPCs [35]. However, this population group has unique characteristics, thus such studies will not be eligible for inclusion.

We will include studies that report on health-care providers' experiences including perspectives on the challenges they face around the pathways to diagnosis of a SPC. Exclusions will be made for studies that report on pathways to diagnosis for recurrent first primary cancers or metastatic cancers and their associated patient and provider experiences.

For peer review only

We will consider studies from any country conducted in primary, secondary or tertiary setting that are focussed on the pathways to diagnosis for a SPC. Furthermore, experiences of health-care providers around the pathways to diagnosis for a SPC in any setting will be eligible for inclusion.

Eligible study designs include observational studies such as cohorts, case control and cross sectional studies that report on the pathways to diagnosis for any type of SPC. Systematic reviews, mixed methods and qualitative studies such as interviews, focus group discussions and ethnography reporting on the pathways to diagnosis for any SPC and associated patient and health-care provider experiences will also be eligible for inclusion. It is unlikely that study designs such as randomised controlled trials (RCTs) will describe the pathways to diagnosis for a SPC and/or patient and provider experiences along that pathway as they are often focussed on interventions, thus, such studies will be excluded. Furthermore, conference abstracts, case reports, book chapters and studies that use secondary datasets that are not focussed on the diagnostic pathways for a SPC or patient/ provider experiences will be excluded. In addition, if these studies do not have complete study methodologies, findings and discussion sections, they will also be excluded from the review.

Search strategy

We will search published and unpublished literature with no restrictions on the date or language to minimise selection bias. A search strategy of relevant Medical Sub-headings [MeSH] terms was designed, piloted and then further refined [Table 1].

To identify relevant published studies, the search strategy will be run initially in Medline through the OVID platform. It will then be adapted and suitable changes made before re-running it again in Embase, CAB Abstracts, Medline-in-process and non-indexed citations, PsycInfo and Epub Ahead of Print, in-process & other non-indexed citations, Ovid Medline Daily. Other sources such as CINAHL, ASSIA, Sociological Abstracts, Web of Science and PROSEPRO will also be searched and the search

For peer review only

strategy adapted accordingly. References lists of relevant studies identified will also be scrutinised for any potential additional studies.

To identify relevant studies from unpublished literatures, we will search grey literature in OpenGrey, ProQuest Dissertations and Theses Global databases for reports and theses.

Screening and data extraction

All the citations found from the various databases will be imported into Endnote X7. Independently, two reviewers (LK and AB) will screen the titles and abstracts against the inclusion and exclusion criteria. Another two reviewers (CC and DC) will independently screen a minimum 10% of these titles and abstracts to check for consistency. Full texts of all potentially relevant articles will be retrieved and again screened by at least two reviewers independently to ascertain whether they have met the inclusion - exclusion criteria. Any disagreements will be resolved through team discussions.

A data extraction form will be designed and piloted to ensure that it captures all the relevant data. The included studies will be divided between reviewers, where one reviewer will carry out the data extraction of the first half and the second reviewer carry out the data extraction of the second half. Both reviewers will do this independently, and then countercheck each other's data extraction.

The data extraction form will include key characteristics of the studies such as author, publication year, data source, country of study, study design and setting (i.e. primary, secondary or tertiary care). In addition, characteristics of the study population such age, gender, ethnicity and cancer types will be included which will provide the context for explanations and interpretations. Any information on the pathways to diagnosis for a SPC will be extracted. This includes; routes to diagnosis, the onset of first symptoms, first presentation or first contact with the healthcare provider, first investigation of cancer related symptoms, referral to hospital, first specialist visit, diagnosis and start of treatment. Furthermore, information on appraisal, help-seeking, diagnostic

For peer review only - http://bmjopen.bmj.com/site/about/guidelines.xhtml

and pre-treatment intervals will also be extracted. Any other relevant patient and provider experiences (including challenges) for SPCs along the diagnostic pathway will also be extracted.

Quality assessment

Quality assessment will be carried out in parallel to data extraction. The quality assessment tool will vary depending on the study design of the included article. There is currently no consensus as to which instrument is 'best' for assessing observational studies, especially of non-intervention studies. Therefore, we plan to adapt several published tools as shown in Table 3. We will use the Critical Appraisal Skills Programme (CASP) tool for systematic reviews, case control and cohort studies. The Critical Appraisal Skills Programme [CASP] tool for qualitative studies will also be used. Within the literature on synthesis of qualitative studies it has been argued that appraisal tools focus more on the methodological rather than the conceptual strengths [36,37]. Therefore, in order to ensure that the authors' theoretical insights are captured, qualitative studies will not be excluded because of the quality of the methods alone.

The McMaster Critical Review tool will be adapted for cross sectional and before after studies guided by the STROBE statement [38-40]. The Mixed Methods Appraisal Tool [MMAT] developed by the National Collaborating Centre for Methods and Tools at McMaster University will be used for mixed methods studies [41].

We will also apply the Aarhus checklist as a framework for describing the rigour of papers to studies that describe and measure intervals on the pathways to cancer diagnosis for a SPC. The Aarhus checklist was developed as a resource for early diagnosis research that describes and measures intervals in patient cancer journeys [33].

For peer review only

Data synthesis

We are uncertain of the profile of study types that we will uncover, although we anticipate most will be descriptive studies. As such, data synthesis will depend on the type of papers identified. Tables and diagrams or charts will be used to summarise the findings as appropriate.

For the qualitative studies, we anticipate that the available data will be limited (as informed by an initial scoping exercise) and as such, meta-ethnography analysis will not be appropriate. Therefore, a narrative framework will be used to summarise and describe the available evidence of pathways to diagnosis of a SPC[42]. Similarly, narrative synthesis will be used to summarise findings from quantitative studies. It is unlikely that a meta-analysis will be applicable because the scope of this review is not intended to cover survival outcomes along the diagnostic pathway for SPCs. Where information on routes to detection is available these will be analysed separately.

Increasingly, narrative synthesis in systematic reviews has been used as a way of collating evidence from research that has been conducted by a range of methods [43]. Although it has been criticised for its lack of transparency, we plan to be explicit in how we identify key concepts and themes across individual studies. The two models of pathways to cancer diagnosis mentioned earlier, the pathways to treatment model [24], and the Olesen model of time intervals [32] as adapted by Weller et al [33] will guide the synthesis.

We will first start by developing a preliminary synthesis, and exploring the relationships within and across studies. NVIVO 10 will facilitate the process of comparing and contrasting information across the included studies where possible, which will allow for indexing and coding of themes to be done systematically. The focus of the synthesis will be to map out a comparison of the included studies against the model of pathways to treatment and the model of time intervals. It is anticipated that this comparison will lead to identification of key findings of similarities and differences within and across the included studies.

For peer review only

Ethics and dissemination

Research Ethics approval was not applicable for this systematic literature review. The University of Edinburgh Centre for Population Health Sciences Ethical Review Committee reviewed and approved the study. We anticipate our findings will help provide a more complete picture of the diagnostic pathway for SPCs, with the potential to inform policy and consistent practice. An understanding of the timing and reasons behind the decision by these patients to seek care is vital if we are to develop interventions to reduce diagnostic delays in this important sub-group of cancer patients. The results will be published in peer-reviewed literature, and presented at relevant cancer and primary care conferences.

For peer review only

Acknowledgements

The systematic review is part of a larger study looking at understanding pathways to diagnosis, patient and providers' experiences and survival outcomes for second primary cancers, funded by the Cancer Research UK (Reference C12357/A21326). The team would like to express their sincere thanks and gratitude to Marshall Dozier for her advice on the searches and Endnote.

Authors' contributions

The systematic review was conceived by DC, KB, CC and DW. LK and AB designed the systematic review protocol with inputs from DC, CC, DW, DHB and KB; LK drafted the manuscript with subsequent input from other authors.

Funding

This work is supported by the Cancer Research UK, reference code A21326

Competing interests

None to declare

For peer review only

References

1. Ahmad, A.S., Ormiston-Smith, N., Sasieni P.D., Trends in the lifetime risk of developing cancer in Great Britain: comparison of risk for those born from 1930 to 1960. Br J Cancer, 2015. 112(5): p. 943-7.

2. Wood, M.E., Vogel, V., Ng, A., Foxhall, L., Goodwin, P., Travis, L., Second malignant neoplasms: assessment and strategies for risk reduction. J Clin Oncol, 2012. 30(30): p. 3734-45

3. National Cancer Institute, U.S. Second primary cancer., Department of Health and Human Services Available at: https://www.cancer.gov/publications/dictionaries/cancer-terms?cdrid=46658 [accessed 13/10/2016]

4. Coyte, A., Morrison, D.S., McLoone, P., Second primary cancer risk - the impact of applying different definitions of multiple primaries: results from a retrospective population based cancer registry study. BMC cancer, 2014. 14: p. 272.

5. Department of Health, M.S.N.I., Living with & Beyond Cancer: Taking Action to Improve Outcomes (an update to the 2010 The National Cancer Survivorship Initiative Vision). March 2013': London SE1.

6. Mellemkjaer, L., Friis, S., Olsen, J., Scelo, G., Hemminki, K., Tracey, E et al., Risk of second cancer among women with breast cancer. Int J Cancer, 2006. 118(9): p. 2285-92.

7. Evans, H.S., Moller, H., Robinson, D., Lewis, C.M., Bell, C.M.J., Hodgson, S.V., The risk of subsequent primary cancers after colorectal cancer in southeast England. Gut, 2002. 50(5): p. 647-52.

For peer review only

8. Hayat, M.J., Howlader, N., Reichman M.E., Edwards, B.K., Cancer statistics, trends, and multiple primary cancer analyses from the Surveillance, Epidemiology, and End Results (SEER) Program. The oncologist, 2007. 12(1): p. 20-37.

9. Phipps, A.I., Chan, A.T., Ogino, S., Anatomic sub-site of primary colorectal cancer and subsequent risk and distribution of second cancers. Cancer, 2013. 119(17): p. 3140-7.

10. Youlden, D.R. and P.D. Baade., The relative risk of second primary cancers in Queensland, Australia: a retrospective cohort study. BMC cancer, 2011. 11: p. 83.

11. Molina-Montes, E., Requena, M., Sanchez-Cantalejo, E., Fernandez, M., Arroyo-Morales, M., Espin, J. et al., Risk of second cancers cancer after a first primary breast cancer: a systematic review and meta-analysis. Gynecologic oncology, 2015. 136(1): p. 158-71.

12. Utada, M., Ohno, Y., Hori, M., Soda, M., Incidence of multiple primary cancers and interval between first and second primary cancers. Cancer science, 2014. 105(7): p. 890-6.

13. Rennemo, E., Zatterstrom, U., Boysen, M., Impact of second primary tumors on survival in head and neck cancer: an analysis of 2,063 cases. Laryngoscope, 2008. 118(8): p. 1350-6.

14. Travis, L.B., The epidemiology of second primary cancers. Cancer Epidemiol Biomarkers Prev, 2006. 15(11): p. 2020-6.

15. Oeffinger, K.C., Baxi, S.S., Friedman, N.D., Moskowitz, C.S., Solid tumor second primary neoplasms: who is at risk, what can we do? Semin Oncol, 2013. 40(6): p. 676-89.

16. Ries, L.A.G., Eisner, M.P., Kosary, C.L., Hankey, B.F., Miller, B.A., Clegg, L., et al., SEER Cancer Statistics Review, 1975-2000, National Cancer Institute. Bethesda (MD): National Cancer Institute, 2003.

For peer review only

17. Deimling, G.T., Bowman, K.F., Sterns, S., Wagner, L.J., Kahana, B., Cancer-related health worries and psychological distress among older adult, long-term cancer survivors. Psychooncology, 2006. 15(4): p. 306-20.

18. Mullens, A.B., McCaul, K.D., Erickson, S.C., Sandgren, A.K. (2004). Coping after cancer: risk perceptions, worry, and health behaviors among colorectal cancer survivors. Psychooncology. 13, 367-376.

19. Wilkins, K.L. and R.L. Woodgate, Life after cancer: living with risk. Cancer Nurs, 2011. 34(6): p. 487-94

20. Smith, L.K., Pope, C., Botha, J.L., Patients' help-seeking experiences and delay in cancer presentation: a qualitative synthesis. Lancet 2005 vol. 366 (3)

21. Shin, D.W., Baik, W.J., Kim, Y.W., Oh, J.H., Chung, K.W., Kim, S.W., et al., Knowledge, attitudes, and practice on second primary cancer screening among cancer survivors: a qualitative study. Patient Educ Couns, 2011. 85(1): p. 74-8.

22. Hamilton, W., Five misconceptions in cancer diagnosis. Br J Den Practice 2009, 59:441-47

23. Khan, N.F., E. Watson, and P.W. Rose, Primary care consultation behaviours of long-term, adult survivors of cancer in the UK. Br J Gen Pract, 2011. 61(584): p. 197-9.

24. Walter, F., Webster, A., Scott, S., Emery, J., The Andersen Model of Total Patient Delay: a systematic review of its application in cancer diagnosis. J Health Serv Res Policy, 2012. 17(2): p. 110-8.

25. Richards, M.A., Westcombe, A.M., Love, S.B., Littlejohns, P., Ramirez, A.J., Influence of delay on survival in patients with breast cancer: a systematic review. Lancet 1999, 353: 1119-26

For peer review only

26. Sogaard, M., Thomsen, R.W., Bossen, K.S., Sorensen, H.T., Norgaard, M, The impact of comorbidity on cancer survival: a review. Clinical Epidemiology 2013: 5 (Suppl 1) 3 - 29

27. Fisher, D.A., Zullig, L.L., Grambow, S.C., Abbott, D.H., Sandler, R.S., Fletcher, R.H., El-Serag, H.B., Provenzale, D., Determinants of medical system delay in the diagnosis of colorectal cancer within the Veteran Affairs Health System. Dig Dis Sci 2010, 55: 1434 – 1441

28. Jordan, S.J., Francis, J.E., Nelson, A.E., Zorbas, H.M., Luxford, K.A., Webb, P.M., Pathways to the diagnosis of epithelial ovarian cancer in Australia. Med J Aust 2010, 193: 326 – 330

29. Macleod, U., Mitchell, E., Burgess, C., Macdonald, S., Ramirez, A.J., Risk factors for delayed presentation and referral of symptomatic cancer: evidence for common cancers. Br J Cancer 2009, 101: S92–S101

30. Elliss-Brookes, L., McPhail, S., Ives, A., Greenslade, M., Shelton, J., Hiom, S., Richards, M., Routes to diagnosis for cancer - determining the patient journey using multiple routine data sets. Br J Cancer 2012, 107: 1220-1226

31. Abel, G.A., Shelton, J., Johnson, S., Ellis-Brookes, L., Lyratzopoulos, G., Cancer-specific variation in emergency presentation by sex, age and deprivation across 27 common and rarer cancers. Br J Cancer 2015, 112(Suppl 2): S129-S136

32. Olesen, F., Hansen, R.P., Vedsted, P., Delay in diagnosis: the experience in Denmark. Br J Cancer 2009, 101(Suppl 2): S5 – S8

33. Weller, D., Vedsted, P., Rubin, G., Walter, F.M., Emery, J., Scott, S., Campbell, C., et al., The Aarhus statement: improving design and reporting of studies on early cancer diagnosis. British Journal of Cancer 2012, 106: 1262-1267

For peer review only

34. Moher, D., Shamseer, L., Clarke, M., Ghersi, D., Liberati, A., Petticrew, M., et al, Preferred Reporting Items for Systematic Review and Meta-Analysis Protocols (PRISMA-P) 2015 statement. Syst Rev. 2015;4(1):1

35. MacDonald, L., Fryer, C., McBride, M., Rogers, P., Pritchard, S., The need for long-term follow-up of childhood cancer survivors in British Columbia. BC Medical Journal 2010: 52(10)

36. Mays, N., Pope, C., Qualitative research in health care. Assessing quality in qualitative research. BMJ (Clinical research ed). 2000;320(7226):50-2.

37. Dixon-Woods, M., Shaw, R.L., Agarwal, S., Smith, J.A., The problem of appraising qualitative research. Quality & safety in health care. 2004;13(3):223-5.

38. Critical Appraisal Skills Programme (CASP), CASP checklists. CASP UK. Accessed 20/10/2016

39. Law, et al, McMaster Critical Review tool and its guidelines. Available at: https://www.unisa.edu.au/Global/Health/Sansom/Documents/iCAHE/CATs/McMasters_Quantitative%20review.pdf. Accessed 20/10/2016

40. STROBE, Strengthening the reporting of observational studies in epidemiology. ISPM - University of Bern 2009. Accessed 20/10/2016

41. National Collaborating Centre for Methods and Tools. Appraising Qualitative, Quantitative, and Mixed Methods Studies included in Mixed Studies Reviews: The MMAT (2015). Hamilton, ON: McMaster University.

42. Popay, J., Roberts, H., Sowden, A., Petticrew, M., Arai, L., Rodgers, M., Britten, N., Roen, K., Duffy, S. Guidance on the conduct of narrative synthesis in systematic reviews. ESRC Methods Programme (2006). Available at: http://citeseerx.ist.psu.edu/viewdoc/download?doi=10.1.1.178.3100&rep=rep1&type=pdf

For peer review only

43. Rodgers, M., Sowden, A., Petticrew, M., Arai, L., Roberts, H., Britten, N., Popay, J., Testing methodological guidance on the conduct of narrative synthesis in systematic reviews: effectiveness of interventions to promote smoke alarm ownership and function. Evaluation 2009; 15:49–74

For peer review only

Table 1: Example search strategy for MEDLINE(R)

1. exp second primary cancer/
2. second primary cancer$.ti,ab.
3. second primary malignan$.ti,ab.
4. second primary tumo$r$.ti,ab.
5. second primary neoplasm$.ti,ab.
6. or/1-5 [SPC TERMS]

---

7. (patient experience$ or patient know$ or patient perception$ or patient perspective$ or patient view$ or patient interpret$ or patient understand$ or patient aware$ or patient attitude).ti,ab.
8. ((health$care provider$ or health$care personnel or health$care professional$ or health$care practitioner$ or doctor$ or general practitioner$ or GP$ or consultant$) adj2 (experience$ or challenge$ or view$ or understanding$)).ti,ab.
9. or/7-8 [PATIENT & PROVIDER TERMS]

---

10. pathway$ to diagnos$.ti,ab.
11. diagnos$ journey.ti,ab.
12. diagnos$ pathway$.ti.
13. diagnos$ pathway$.ti,ab.
14. (diagnos$ adj2 pathway$).ti,ab.
15. route$ to diagnos$.ti,ab.
16. diagnos$ route$.ti,ab.
17. pathway$ to treatment$.ti,ab.
18. (patient appraisal or help$seeking behavio$r$ or diagnos$ interval$ or pre$treatment interval$).ti,ab.
19. (first symptom or first presentation or clinical appearance or first investigation or first referral or first specialist visit or diagnosis or treatment start).ti,ab.
20. ((patient appraisal and self$ management) or decision to consult health$care provider or health$care provider appraisal, investigations, referrals, appointment$ or planning or scheduling treatment).ti,ab.
21. or/10-20 [PATHWAYS TO DIAGNOSIS TERMS]

---

22. 9 or 21 [PATIENTS & PROVIDER TERMS AND PATHWAYS TO DIAGNOSIS TERMS)

---

23. 6 and 22 [CANCER TERMS AND PATIENTS & PROVIDER TERMS AND PATHWAYS TO DIAGNOSIS TERMS COMBINED]

---

24. limit 23 to humans [LIMITED TO HUMAN ONLY STUDIES]

For peer review only

Table 2: Inclusion – Exclusion criteria

	Inclusion	Exclusion
**Pathways to diagnosis**	- Any stage of the pathway to diagnosis for a SPC<br>- Appraisal, help seeking, diagnostic and pre-treatment intervals<br>- Routes to diagnosis	- Pathways to diagnosis for recurrent first primary cancers or metastatic cancers<br>- Studies focusing only on the time between the diagnosis of a FPC and SPC
**Patients**	- SPCs in patients above 18 years of age<br>- Patients diagnosed with first primary cancer at 18 years and above	- SPCs in children and adolescents<br>- SPCs in adults following a childhood or adolescent first primary cancer
**Health-care providers**	- Health-care providers' involved in the diagnosis and management of patients with SPC in the diagnostic pathway	-
**Key outcomes of interest**	- Pathways to diagnosis for SPCs as per the inclusion criteria above<br>- How patients appraise, interpret symptoms, and engage with healthcare in the diagnosis of a SPC<br>- Health-care providers' experiences, perspectives and challenges faced around the pathways to diagnosis of a SPC	- Patient and health-care provider experiences related to pathways to diagnosis for recurrent first primary cancers or metastatic cancers
**Setting**	- Primary, secondary or tertiary care in any country	-
**Study designs**	- Cohorts, case control and cross sectional studies<br>- Systematic reviews and mixed methods<br>- Qualitative studies such as interviews, focus group discussions and ethnography on the pathways to diagnosis for any SPC and associated patient and health-care provider experiences	- RCTs<br>- Studies using secondary datasets or are conference abstracts, conference proceedings and book chapters that are not focussed on the diagnostic pathways for a SPC or patient/ provider experiences of SPCs<br>- Case reports

For Peer Review Only

Table 3: Quality Assessment tools

Tools	Study designs
CASP checklist	Systematic review<br>Case control<br>Cohorts<br>Qualitative [e.g. interviews, focus group discussions and ethnography]
McMaster Critical Review Tool & STROBE	Cross-sectional<br>Before and after
MMAT	Mixed methods
Aarhus checklist	All included studies that report and measure intervals of patient cancer journey

For peer review only

Figure 1: Pathways to treatment model [24]

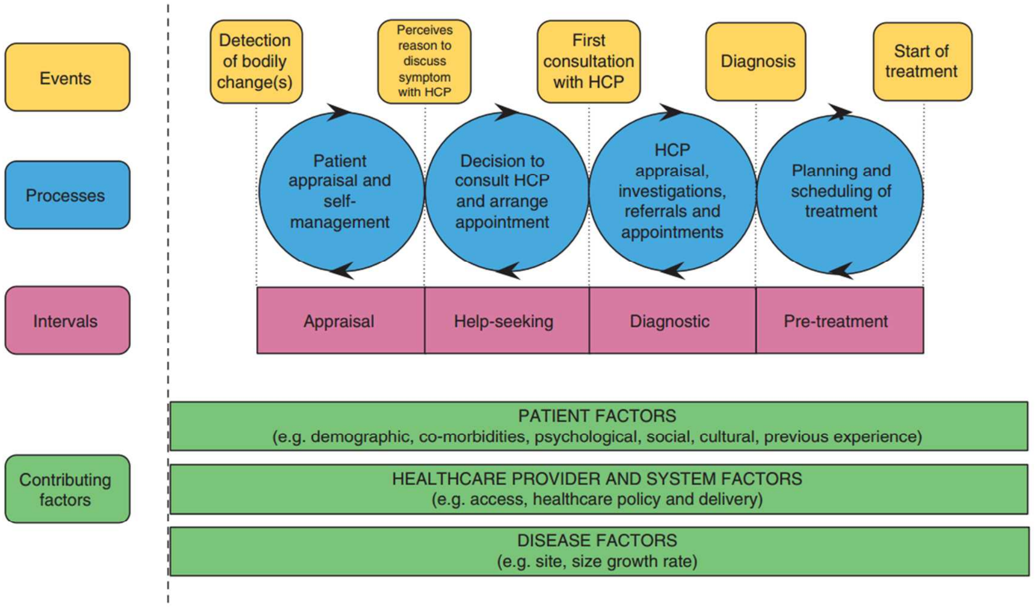

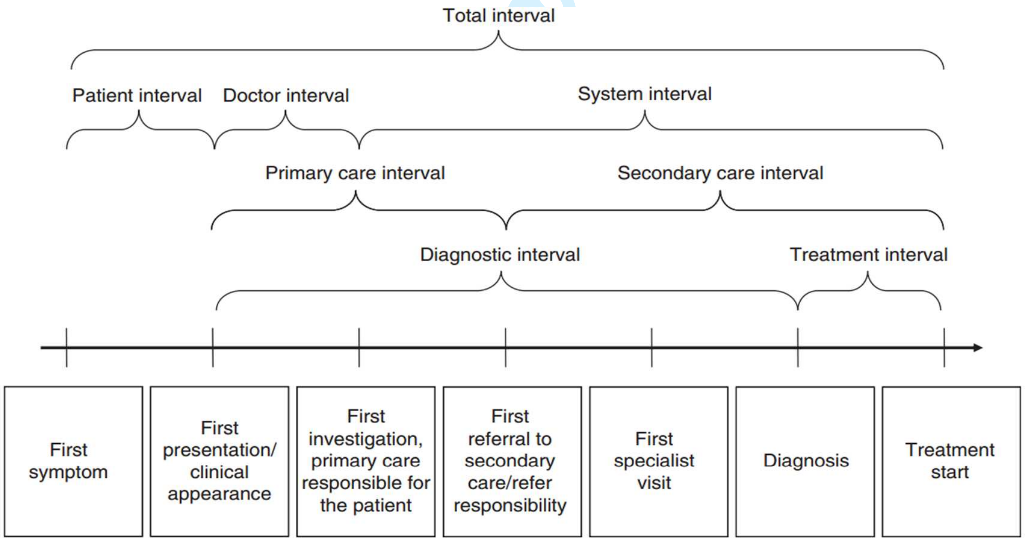

Figure 2: Model of key time points and intervals [[32] as adapted by Weller et al [33]]

PRISMA 2009 Checklist

Section/topic	#	Checklist item	Reported on page #
**TITLE**			
Title	1	Identify the report as a systematic review, meta-analysis, or both.	1
**ABSTRACT**			
Structured summary	2	Provide a structured summary including, as applicable: background; objectives; data sources; study eligibility criteria, participants, and interventions; study appraisal and synthesis methods; results; limitations; conclusions and implications of key findings; systematic review registration number.	2
**INTRODUCTION**			
Rationale	3	Describe the rationale for the review in the context of what is already known.	4-5
Objectives	4	Provide an explicit statement of questions being addressed with reference to participants, interventions, comparisons, outcomes, and study design (PICOS).	5
**METHODS**			
Protocol and registration	5	Indicate if a review protocol exists, if and where it can be accessed (e.g., Web address), and, if available, provide registration information including registration number.	2
Eligibility criteria	6	Specify study characteristics (e.g., PICOS, length of follow-up) and report characteristics (e.g., years considered, language, publication status) used as criteria for eligibility, giving rationale.	6 - 7
Information sources	7	Describe all information sources (e.g., databases with dates of coverage, contact with study authors to identify additional studies) in the search and date last searched.	7
Search	8	Present full electronic search strategy for at least one database, including any limits used, such that it could be repeated.	19
Study selection	9	State the process for selecting studies (i.e., screening, eligibility, included in systematic review, and, if applicable, included in the meta-analysis).	8
Data collection process	10	Describe method of data extraction from reports (e.g., piloted forms, independently, in duplicate) and any processes for obtaining and confirming data from investigators.	8
Data items	11	List and define all variables for which data were sought (e.g., PICOS, funding sources) and any assumptions and simplifications made.	8
Risk of bias in individual studies	12	Describe methods used for assessing risk of bias of individual studies (including specification of whether this was done at the study or outcome level), and how this information is to be used in any data synthesis.	10
Summary measures	13	State the principal summary measures (e.g., risk ratio, difference in means).	N/A
Synthesis of results	14	Describe the methods of handling data and combining results of studies, if done, including measures of consistency (e.g., I^2) for each meta-analysis.	N/A

For peer review only - http://bmjopen.bmj.com/site/about/guidelines.xhtml

PRISMA 2009 Checklist

Section/topic	#	Checklist item	Reported on page #
Risk of bias across studies	15	Specify any assessment of risk of bias that may affect the cumulative evidence (e.g., publication bias, selective reporting within studies).	9
Additional analyses	16	Describe methods of additional analyses (e.g., sensitivity or subgroup analyses, meta-regression), if done, indicating which were pre-specified.	10
**RESULTS**			
Study selection	17	Give numbers of studies screened, assessed for eligibility, and included in the review, with reasons for exclusions at each stage, ideally with a flow diagram.	N/A
Study characteristics	18	For each study, present characteristics for which data were extracted (e.g., study size, PICOS, follow-up period) and provide the citations.	N/A
Risk of bias within studies	19	Present data on risk of bias of each study and, if available, any outcome level assessment (see item 12).	N/A
Results of individual studies	20	For all outcomes considered (benefits or harms), present, for each study: (a) simple summary data for each intervention group (b) effect estimates and confidence intervals, ideally with a forest plot.	N/A
Synthesis of results	21	Present results of each meta-analysis done, including confidence intervals and measures of consistency.	N/A
Risk of bias across studies	22	Present results of any assessment of risk of bias across studies (see Item 15).	N/A
Additional analysis	23	Give results of additional analyses, if done (e.g., sensitivity or subgroup analyses, meta-regression [see Item 16]).	N/A
**DISCUSSION**			
Summary of evidence	24	Summarize the main findings including the strength of evidence for each main outcome; consider their relevance to key groups (e.g., healthcare providers, users, and policy makers).	N/A
Limitations	25	Discuss limitations at study and outcome level (e.g., risk of bias), and at review-level (e.g., incomplete retrieval of identified research, reporting bias).	N/A
Conclusions	26	Provide a general interpretation of the results in the context of other evidence, and implications for future research.	N/A
**FUNDING**			
Funding	27	Describe sources of funding for the systematic review and other support (e.g., supply of data); role of funders for the systematic review.	12

From: Moher D, Liberati A, Tetzlaff J, Altman DG, The PRISMA Group (2009). Preferred Reporting Items for Systematic Reviews and Meta-Analyses: The PRISMA Statement. PLoS Med 6(7): e1000097. doi:10.1371/journal.pmed1000097

For more information, visit: www.prisma-statement.org.

For peer review only - http://bmjopen.bmj.com/site/about/guidelines.xhtml

BMJ Open

Pathways to diagnosis of a second primary cancer: protocol for a mixed methods systematic review

Journal:	*BMJ Open*
Manuscript ID	bmjopen-2017-017929.R1
Article Type:	Protocol
Date Submitted by the Author:	25-Jul-2017
Complete List of Authors:	Kanguru, Lovney; University of Edinburgh, Usher Institute of Population Heath Sciences and Informatics<br>Bikker, Annemieke; University of Edinburgh, Usher Institute of Population Heath Sciences and Informatics<br>Cavers, Debbie; University of Edinburgh, Usher Institute of Population Heath Sciences and Informatics<br>Barnett, Karen; University of Edinburgh, Usher Institute of Population Heath Sciences and Informatics<br>Brewster, David; Scottish Cancer Registry<br>Weller, David; University of Edinburgh, Usher Institute of Population Heath Sciences and Informatics<br>Campbell, Christine; University of Edinburgh, Usher Institute of Population Heath Sciences and Informatics
**Primary Subject Heading**:	Public health
Secondary Subject Heading:	Public health, Oncology, Health services research
Keywords:	Second primary cancer, diagnostic pathway, routes to diagnosis, diagnostic interval, patient and health-care provider experiences, heath-care provider challenges

SCHOLARONE™
Manuscripts

Title

Pathways to diagnosis of a second primary cancer: protocol for a mixed methods systematic review

Authors

Lovney Kanguru[1*], Annemieke Bikker[1], Debbie Cavers[1], Karen Barnett[1], David H Brewster [1, 2], David Weller[1], Christine Campbell[1]

Affiliation

[1]Usher Institute of Population Heath Sciences and Informatics, University of Edinburgh, EH8 9AG

[2]Scottish Cancer Registry, Information Services Division, NHS National Services Scotland, Edinburgh EH12 9EB

***Corresponding Author**

Lovney.Kanguru@ed.ac.uk

For peer review only

Abstract

Introduction: As cancer survivors continue to live longer, the incidence of second primary cancers (SPCs) will also rise. Relatively little is understood about the diagnostic pathway for SPCs, how people appraise, interpret symptoms and seek help for a second different cancer, and the experiences (including challenges) of health-care providers relating to SPCs. This study aims to systematically appraise and synthesise the literature on the pathways to diagnosis of a SPC and the associated patient and health-care provider experiences.

Methods: The approach taken includes systematic searches of published and unpublished literature without any date or language restrictions. Medline, Embase, CAB Abstracts, Medline-in-process and non-indexed citations, PsycInfo, Epub Ahead of Print in-process & other non-indexed citations Ovid Medline Daily, CINAHL, ASSIA, Sociological Abstracts, Web of Science, PROSPERO and grey literature will be searched to identify observational, systematic reviews, mixed methods and qualitative studies of interest. Titles, abstracts and full texts will be screened against the inclusion - exclusion criteria by at least two reviewers independently. Relevant outcomes of interest and study and population characteristics will be extracted. Synthesis will be used guided by the pathways to treatment model, and the Olesen model of time intervals.

Ethics and dissemination: Ethical approval is not required. This systematic review will provide a deeper understanding of the complex and heterogeneous diagnostic pathways of SPCs, while identifying common themes across the diagnostic interval, routes to diagnosis and patient and health-care provider experiences. These findings will help provide a nuanced picture of the diagnostic pathway for SPCs that may inform policy and consistent practice in particular approaches to early diagnosis for a SPC including the timing and reasons behind the decision by the patient to seek care, challenges faced by health-care providers, and in the development of future interventions to reduce the delay in patient time-to-presentation.

Registration: PROSPERO CRD42016051692

For peer review only

Strengths and limitations of this study

- This is the first systematic review to appraise and synthesise the literature on the pathways to diagnosis of a SPC and the associated patient and health-care provider experiences
- This study may help provide a nuanced picture of the diagnostic pathway for SPCs that may inform policy and consistent practice in particular approaches to early diagnosis for a SPC including the timing and reasons behind the decision by the patient to seek care, challenges faced by health-care providers, and in the development of future interventions
- The review is not restricted to any time period or specific languages
- We anticipate that the available data from qualitative studies may be limited (as informed by an initial scoping exercise) and as such, meta-ethnography synthesis may not be possible to do
- It is unlikely that a meta-analysis will be applicable for the quantitative studies included because the scope of this review does not cover survival outcomes along the diagnostic pathway for SPCs

For peer review only

Introduction

The number of people surviving cancer is steadily increasing [1]. As cancer survivors continue to live longer, in common with the wider population, the incidence of second primary cancers (SPCs) is also expected to grow [2]. A second primary cancer has been defined as a new primary cancer that occurs in a person who has had cancer in the past [3]. Evidence suggests that the incidence of SPCs among cancer survivors ranges between 1% and 17% depending on the index cancer site [4-13], with SPCs estimated to account for as much as 16-18% of total cancer incidence [14-16] in western countries.

Fear of a cancer recurring and the possibility of getting a new primary cancer or multiple primaries are often a source of worry and psychosocial distress among cancer survivors [17-19]. These cancer-related health worries have been linked to higher levels of mental illness particularly anxiety and depression in long-term survivors [17]. Furthermore, the fear of, and past experiences of cancer have been shown to affect patients' help seeking behaviour [20-23]. Symptoms of a SPC may be missed if patients are not aware of their significance [20]. This could be because of patients focusing on the symptoms of the original cancer only, or patients' perceptions being influenced by the presence of co-morbid conditions [24, 25, 26].

Early detection of cancers is an important element in improving cancer outcomes, as the stage of disease at diagnosis is linked to survival for many cancers [20, 25]. While patients' ability to recognise possible warning signs and taking prompt action is a critical early step, evidence suggests that the patient diagnostic pathway is often not straightforward [27 - 31]. For example, the diagnostic interval may vary depending on the route to diagnosis, which has potential implications for the stage at diagnosis. The Pathways to Treatment Model [24] provides a theoretical framework for the wide range of factors that influence the pathway to diagnosis, not only those relating to the patient, but also those linked to the health-care provider, the health system and the cancer itself [Figure 1]. There are also key time-points in diagnostic journeys (Olesen et al [32]; Weller et al [33]) as illustrated in Figure 2.

For peer review only

Both models emphasise the often-complex journeys that patients go through from the onset of first symptoms to the start of treatment.

A greater understanding of patient pathways to diagnosis (including diagnostic routes) for a SPC is vital due to the growing incidence, yet comparatively little is known about these pathways. Mapping the evidence on pathways to diagnosis for a SPC and the associated patient and health-care providers' experiences will be an important starting point that will provide a nuanced picture of the diagnostic pathway for SPCs. These insights may help to inform policy and consistent practice in approaches to early diagnosis for a SPC, including the timing and the decision by the patient to seek care, and in the development of future interventions to reduce the delay in patient time-to-presentation.

This review aims to systematically appraise and synthesise the literature on pathways to diagnosis of a SPC and associated patient and health-care provider experiences. Our objectives are:

1. To summarise the literature describing pathways to diagnosis of a SPC, including any descriptions of patient and diagnostic intervals, and routes to diagnosis.
2. To summarise the literature describing how patients appraise symptoms and engage with health care in the pathway to diagnosis of a SPC
3. To explore the available evidence on health-care providers' perspectives around the pathways to diagnosis of a SPC and the challenges they face

For peer review only

METHODS

The PRISMA-P statement [34] has guided the development and reporting of this systematic review protocol.

Inclusion and exclusion criteria

Table 1 summarises the inclusion – exclusion criteria. This review will consider studies that report on any stage of the pathways to diagnosis for a SPC, including routes to diagnosis (e.g. during routine follow up or surveillance relating to the first cancer, through engagement with screening, symptomatic presentation in general practice or through emergency presentation). We will also consider studies that report on patient and diagnostic intervals such as symptom appraisal, help seeking behaviour, diagnostic and pre-treatment intervals [24, 28, 33]. We will follow the definitions of appraisal, help-seeking, diagnostic and pre-treatment intervals as defined in the Aarhus Statement [33]. Although these definitions are important in this review, we will not exclude studies because of lack of, or poor definitions only.

Studies that report on how patients appraise and interpret symptoms, engage with healthcare in the diagnosis of a SPC, and on routes to diagnosis, will also be included. Studies for inclusion will be limited to those that examine patients with SPCs only and where patients are 18 years and above at the time of first primary cancer diagnosis. We recognise that SPCs in adults following a childhood or adolescent primary cancer is an important area, and there is substantial work that has been done on late effects of childhood cancers that includes SPCs [35]. However, this population group has unique characteristics, thus such studies will not be eligible for inclusion.

We will include studies that report on health-care providers' experiences including perspectives on the challenges they face around the pathways to diagnosis of a SPC. Exclusions will be made for studies that report on pathways to diagnosis for recurrent first primary cancers or metastatic cancers and their associated patient and provider experiences.

For peer review only

We will consider studies from any country conducted in primary, secondary or tertiary setting that are focussed on the pathways to diagnosis for a SPC. Furthermore, experiences of health-care providers around the pathways to diagnosis for a SPC in any setting will be eligible for inclusion.

Eligible study designs include observational studies such as cohorts, case control and cross sectional studies that report on the pathways to diagnosis for any type of SPC. Systematic reviews, mixed methods and qualitative studies such as interviews, focus group discussions and ethnography reporting on the pathways to diagnosis for any SPC and associated patient and health-care provider experiences will also be eligible for inclusion. It is unlikely that study designs such as randomised controlled trials (RCTs) will describe the pathways to diagnosis for a SPC and/or patient and provider experiences along that pathway as they are often focussed on interventions, thus, such studies will be excluded. Furthermore, conference abstracts, case reports, book chapters and studies that use secondary datasets that are not focussed on the diagnostic pathways for a SPC or patient/ provider experiences will be excluded. In addition, if these studies do not have complete study methodologies, findings and discussion sections, they will also be excluded from the review.

Search strategy

We will search published and unpublished literature with no restrictions on the date or language to minimise selection bias. A search strategy of relevant Medical Sub-headings [MeSH] terms was designed, piloted and then further refined [Table 2].

To identify relevant published studies, the search strategy will be run initially in Medline through the OVID platform. It will then be adapted and suitable changes made before re-running it again in Embase, CAB Abstracts, Medline-in-process and non-indexed citations, PsycInfo and Epub Ahead of Print, in-process & other non-indexed citations, Ovid Medline Daily. Other sources such as CINAHL, ASSIA, Sociological Abstracts, Web of Science and PROSEPRO will also be searched and the search

For peer review only

strategy adapted accordingly. References lists of relevant studies identified will also be scrutinised for any potential additional studies.

To identify relevant studies from unpublished literatures, we will search grey literature in OpenGrey, ProQuest Dissertations and Theses Global databases for reports and theses.

Screening and data extraction

All the citations found from the various databases will be imported into Endnote X7. Independently, two reviewers (LK and AB) will screen the titles and abstracts against the inclusion and exclusion criteria. Another two reviewers (CC and DC) will independently screen a minimum 10% of these titles and abstracts to check for consistency. Full texts of all potentially relevant articles will be retrieved and again screened by at least two reviewers independently to ascertain whether they have met the inclusion - exclusion criteria. Any disagreements will be resolved through team discussions.

A data extraction form will be designed and piloted to ensure that it captures all the relevant data. The included studies will be divided between reviewers, where one reviewer will carry out the data extraction of the first half and the second reviewer carry out the data extraction of the second half. Both reviewers will do this independently, and then countercheck each other's data extraction.

The data extraction form will include key characteristics of the studies such as author, publication year, period of the study (by year(s)), data source, country of study, study design and setting (i.e. primary, secondary or tertiary care) and type of healthcare system including first point of contact with the healthcare provider. In addition, characteristics of the study population such age, gender, ethnicity and cancer types will be included which will provide the context for explanations and interpretations. Any information on the pathways to diagnosis for a SPC will be extracted. This includes; routes to diagnosis, the onset of first symptoms, first presentation or first contact with the healthcare provider, first investigation of cancer related symptoms, referral to hospital, first specialist visit, diagnosis and start of treatment. Furthermore, information on appraisal, help-

For peer review only

seeking, diagnostic and pre-treatment intervals will also be extracted. Any other relevant patient and provider experiences (including challenges) for SPCs along the diagnostic pathway will also be extracted.

Quality assessment

Quality assessment will be carried out in parallel to data extraction. The quality assessment tool will vary depending on the study design of the included article. There is currently no consensus as to which instrument is 'best' for assessing observational studies, especially of non-intervention studies. Therefore, we plan to adapt several published tools as shown in Table 3. We will use the Critical Appraisal Skills Programme (CASP) tool for systematic reviews, case control and cohort studies. The Critical Appraisal Skills Programme [CASP] tool for qualitative studies will also be used. Within the literature on synthesis of qualitative studies it has been argued that appraisal tools focus more on the methodological rather than the conceptual strengths [36,37]. Therefore, in order to ensure that the authors' theoretical insights are captured, qualitative studies will not be excluded because of the quality of the methods alone.

The McMaster Critical Review tool will be adapted for cross sectional and before after studies guided by the STROBE statement [38-40]. The Mixed Methods Appraisal Tool [MMAT] developed by the National Collaborating Centre for Methods and Tools at McMaster University will be used for mixed methods studies [41].

We will also apply the Aarhus checklist as a framework for describing the rigour of papers to studies that describe and measure intervals on the pathways to cancer diagnosis for a SPC. The Aarhus checklist was developed as a resource for early diagnosis research that describes and measures intervals in patient cancer journeys [33].

For peer review only - http://bmjopen.bmj.com/site/about/guidelines.xhtml

Data synthesis

We are uncertain of the profile of study types that we will uncover, although we anticipate most will be descriptive studies. As such, data synthesis will depend on the type of papers identified. Tables and diagrams or charts will be used to summarise the findings as appropriate. Depending on the included papers, synthesis will consider different categorisations of findings to explore. For example, varying combinations of first primary cancer and SPCs, patterns over time and different healthcare delivery systems.

For the qualitative studies, we anticipate that the available data will be limited (as informed by an initial scoping exercise) and as such, meta-ethnography analysis will not be appropriate. Therefore, a narrative framework will be used to summarise and describe the available evidence of pathways to diagnosis of a SPC[42]. Similarly, narrative synthesis will be used to summarise findings from quantitative studies. It is unlikely that a meta-analysis will be applicable because the scope of this review is not intended to cover survival outcomes along the diagnostic pathway for SPCs. Where information on routes to detection is available, these will be analysed separately.

Increasingly, narrative synthesis in systematic reviews has been used as a way of collating evidence from research that has been conducted by a range of methods [43]. Although it has been criticised for its lack of transparency, we plan to be explicit in how we identify key concepts and themes across individual studies. The two models of pathways to cancer diagnosis mentioned earlier, the pathways to treatment model [24], and the Olesen model of time intervals [32] as adapted by Weller et al [33] will guide the synthesis.

We will first start by developing a preliminary synthesis, and exploring the relationships within and across studies. NVIVO 10 will facilitate the process of comparing and contrasting information across the included studies where possible, which will allow for indexing and coding of themes to be done

For peer review only

systematically. The focus of the synthesis will be to map out a comparison of the included studies against the model of pathways to treatment and the model of time intervals. It is anticipated that this comparison will lead to identification of key findings of similarities and differences within and across the included studies.

Ethics and dissemination

Research Ethics approval was not applicable for this systematic literature review. The University of Edinburgh Centre for Population Health Sciences Ethical Review Committee reviewed and approved the study. We anticipate our findings will help provide a more complete picture of the diagnostic pathway for SPCs, with the potential to inform policy and consistent practice. An understanding of the timing and reasons behind the decision by these patients to seek care is vital if we are to develop interventions to reduce diagnostic delays in this important sub-group of cancer patients. The results will be published in peer-reviewed literature, and presented at relevant cancer and primary care conferences.

For peer review only

Acknowledgements

The systematic review is part of a larger study looking at understanding pathways to diagnosis, patient and providers' experiences and survival outcomes for second primary cancers, funded by the Cancer Research UK (Reference C12357/A21326). The team would like to express their sincere thanks and gratitude to Marshall Dozier for her advice on the searches and Endnote.

Authors' contributions

The systematic review was conceived by DC, KB, CC and DW. LK and AB designed the systematic review protocol with inputs from DC, CC, DW, DHB and KB; LK drafted the manuscript with subsequent input from other authors. CC is the guarantor and the principal investigator of this review.

Funding

This work is supported by the Cancer Research UK, reference code A21326. The funders had no role in the design, decision to publish and preparation of this manuscript.

Competing interests

None to declare

For peer review only

References

1. Ahmad, A.S., Ormiston-Smith, N., Sasieni P.D., Trends in the lifetime risk of developing cancer in Great Britain: comparison of risk for those born from 1930 to 1960. Br J Cancer, 2015. 112(5): p. 943-7.

2. Wood, M.E., Vogel, V., Ng, A., Foxhall, L., Goodwin, P., Travis, L., Second malignant neoplasms: assessment and strategies for risk reduction. J Clin Oncol, 2012. 30(30): p. 3734-45

3. National Cancer Institute, U.S. Second primary cancer., Department of Health and Human Services Available at: https://www.cancer.gov/publications/dictionaries/cancer-terms?cdrid=46658 [accessed 13/10/2016]

4. Coyte, A., Morrison, D.S., McLoone, P., Second primary cancer risk - the impact of applying different definitions of multiple primaries: results from a retrospective population based cancer registry study. BMC cancer, 2014. 14: p. 272.

5. Department of Health, M.S.N.I., Living with & Beyond Cancer: Taking Action to Improve Outcomes (an update to the 2010 The National Cancer Survivorship Initiative Vision). March 2013': London SE1.

6. Mellemkjaer, L., Friis, S., Olsen, J., Scelo, G., Hemminki, K., Tracey, E et al., Risk of second cancer among women with breast cancer. Int J Cancer, 2006. 118(9): p. 2285-92.

7. Evans, H.S., Moller, H., Robinson, D., Lewis, C.M., Bell, C.M.J., Hodgson, S.V., The risk of subsequent primary cancers after colorectal cancer in southeast England. Gut, 2002. 50(5): p. 647-52.

For peer review only

8. Hayat, M.J., Howlader, N., Reichman M.E., Edwards, B.K., Cancer statistics, trends, and multiple primary cancer analyses from the Surveillance, Epidemiology, and End Results (SEER) Program. The oncologist, 2007. 12(1): p. 20-37.

9. Phipps, A.I., Chan, A.T., Ogino, S., Anatomic sub-site of primary colorectal cancer and subsequent risk and distribution of second cancers. Cancer, 2013. 119(17): p. 3140-7.

10. Youlden, D.R. and P.D. Baade., The relative risk of second primary cancers in Queensland, Australia: a retrospective cohort study. BMC cancer, 2011. 11: p. 83.

11. Molina-Montes, E., Requena, M., Sanchez-Cantalejo, E., Fernandez, M., Arroyo-Morales, M., Espin, J. et al., Risk of second cancers cancer after a first primary breast cancer: a systematic review and meta-analysis. Gynecologic oncology, 2015. 136(1): p. 158-71.

12. Utada, M., Ohno, Y., Hori, M., Soda, M., Incidence of multiple primary cancers and interval between first and second primary cancers. Cancer science, 2014. 105(7): p. 890-6.

13. Rennemo, E., Zatterstrom, U., Boysen, M., Impact of second primary tumors on survival in head and neck cancer: an analysis of 2,063 cases. Laryngoscope, 2008. 118(8): p. 1350-6.

14. Travis, L.B., The epidemiology of second primary cancers. Cancer Epidemiol Biomarkers Prev, 2006. 15(11): p. 2020-6.

15. Oeffinger, K.C., Baxi, S.S., Friedman, N.D., Moskowitz, C.S., Solid tumor second primary neoplasms: who is at risk, what can we do? Semin Oncol, 2013. 40(6): p. 676-89.

16. Ries, L.A.G., Eisner, M.P., Kosary, C.L., Hankey, B.F., Miller, B.A., Clegg, L., et al., SEER Cancer Statistics Review, 1975-2000, National Cancer Institute. Bethesda (MD): National Cancer Institute, 2003.

For peer review only

17. Deimling, G.T., Bowman, K.F., Sterns, S., Wagner, L.J., Kahana, B., Cancer-related health worries and psychological distress among older adult, long-term cancer survivors. Psychooncology, 2006. 15(4): p. 306-20.

18. Mullens, A.B., McCaul, K.D., Erickson, S.C., Sandgren, A.K. (2004). Coping after cancer: risk perceptions, worry, and health behaviors among colorectal cancer survivors. Psychooncology. 13, 367-376.

19. Wilkins, K.L. and R.L. Woodgate, Life after cancer: living with risk. Cancer Nurs, 2011. 34(6): p. 487-94

20. Smith, L.K., Pope, C., Botha, J.L., Patients' help-seeking experiences and delay in cancer presentation: a qualitative synthesis. Lancet 2005 vol. 366 (3)

21. Shin, D.W., Baik, W.J., Kim, Y.W., Oh, J.H., Chung, K.W., Kim, S.W., et al., Knowledge, attitudes, and practice on second primary cancer screening among cancer survivors: a qualitative study. Patient Educ Couns, 2011. 85(1): p. 74-8.

22. Hamilton, W., Five misconceptions in cancer diagnosis. Br J Den Practice 2009, 59:441-47

23. Khan, N.F., E. Watson, and P.W. Rose, Primary care consultation behaviours of long-term, adult survivors of cancer in the UK. Br J Gen Pract, 2011. 61(584): p. 197-9.

24. Walter, F., Webster, A., Scott, S., Emery, J., The Andersen Model of Total Patient Delay: a systematic review of its application in cancer diagnosis. J Health Serv Res Policy, 2012. 17(2): p. 110-8.

25. Richards, M.A., Westcombe, A.M., Love, S.B., Littlejohns, P., Ramirez, A.J., Influence of delay on survival in patients with breast cancer: a systematic review. Lancet 1999, 353: 1119-26

For peer review only

26. Sogaard, M., Thomsen, R.W., Bossen, K.S., Sorensen, H.T., Norgaard, M, The impact of comorbidity on cancer survival: a review. Clinical Epidemiology 2013: 5 (Suppl 1) 3 - 29

27. Fisher, D.A., Zullig, L.L., Grambow, S.C., Abbott, D.H., Sandler, R.S., Fletcher, R.H., El-Serag, H.B., Provenzale, D., Determinants of medical system delay in the diagnosis of colorectal cancer within the Veteran Affairs Health System. Dig Dis Sci 2010, 55: 1434 – 1441

28. Jordan, S.J., Francis, J.E., Nelson, A.E., Zorbas, H.M., Luxford, K.A., Webb, P.M., Pathways to the diagnosis of epithelial ovarian cancer in Australia. Med J Aust 2010, 193: 326 – 330

29. Macleod, U., Mitchell, E., Burgess, C., Macdonald, S., Ramirez, A.J., Risk factors for delayed presentation and referral of symptomatic cancer: evidence for common cancers. Br J Cancer 2009, 101: S92–S101

30. Elliss-Brookes, L., McPhail, S., Ives, A., Greenslade, M., Shelton, J., Hiom, S., Richards, M., Routes to diagnosis for cancer - determining the patient journey using multiple routine data sets. Br J Cancer 2012, 107: 1220-1226

31. Abel, G.A., Shelton, J., Johnson, S., Ellis-Brookes, L., Lyratzopoulos, G., Cancer-specific variation in emergency presentation by sex, age and deprivation across 27 common and rarer cancers. Br J Cancer 2015, 112(Suppl 2): S129-S136

32. Olesen, F., Hansen, R.P., Vedsted, P., Delay in diagnosis: the experience in Denmark. Br J Cancer 2009, 101(Suppl 2): S5 – S8

33. Weller, D., Vedsted, P., Rubin, G., Walter, F.M., Emery, J., Scott, S., Campbell, C., et al., The Aarhus statement: improving design and reporting of studies on early cancer diagnosis. British Journal of Cancer 2012, 106: 1262-1267

For peer review only

34. Moher, D., Shamseer, L., Clarke, M., Ghersi, D., Liberati, A., Petticrew, M., et al, Preferred Reporting Items for Systematic Review and Meta-Analysis Protocols (PRISMA-P) 2015 statement. Syst Rev. 2015;4(1):1

35. MacDonald, L., Fryer, C., McBride, M., Rogers, P., Pritchard, S., The need for long-term follow-up of childhood cancer survivors in British Columbia. BC Medical Journal 2010: 52(10)

36. Mays, N., Pope, C., Qualitative research in health care. Assessing quality in qualitative research. BMJ (Clinical research ed). 2000;320(7226):50-2.

37. Dixon-Woods, M., Shaw, R.L., Agarwal, S., Smith, J.A., The problem of appraising qualitative research. Quality & safety in health care. 2004;13(3):223-5.

38. Critical Appraisal Skills Programme (CASP), CASP checklists. CASP UK. Accessed 20/10/2016

39. Law, et al, McMaster Critical Review tool and its guidelines. Available at: https://www.unisa.edu.au/Global/Health/Sansom/Documents/iCAHE/CATs/McMasters_Quantitative%20review.pdf. Accessed 20/10/2016

40. STROBE, Strengthening the reporting of observational studies in epidemiology. ISPM - University of Bern 2009. Accessed 20/10/2016

41. National Collaborating Centre for Methods and Tools. Appraising Qualitative, Quantitative, and Mixed Methods Studies included in Mixed Studies Reviews: The MMAT (2015). Hamilton, ON: McMaster University.

42. Popay, J., Roberts, H., Sowden, A., Petticrew, M., Arai, L., Rodgers, M., Britten, N., Roen, K., Duffy, S. Guidance on the conduct of narrative synthesis in systematic reviews. ESRC Methods Programme (2006). Available at: http://citeseerx.ist.psu.edu/viewdoc/download?doi=10.1.1.178.3100&rep=rep1&type=pdf

For peer review only

43. Rodgers, M., Sowden, A., Petticrew, M., Arai, L., Roberts, H., Britten, N., Popay, J., Testing methodological guidance on the conduct of narrative synthesis in systematic reviews: effectiveness of interventions to promote smoke alarm ownership and function. Evaluation 2009; 15:49–74

For peer review only

Table 1: Inclusion – Exclusion criteria

	Inclusion	Exclusion
**Pathways to diagnosis**	- Any stage of the pathway to diagnosis for a SPC<br>- Appraisal, help seeking, diagnostic and pre-treatment intervals<br>- Routes to diagnosis	- Pathways to diagnosis for recurrent first primary cancers or metastatic cancers<br>- Studies focusing only on the time between the diagnosis of a FPC and SPC
**Patients**	- SPCs in patients above 18 years of age<br>- Patients diagnosed with first primary cancer at 18 years and above	- SPCs in children and adolescents<br>- SPCs in adults following a childhood or adolescent first primary cancer
**Health-care providers**	- Health-care providers' involved in the diagnosis and management of patients with SPC in the diagnostic pathway	-
**Key outcomes of interest**	- Pathways to diagnosis for SPCs as per the inclusion criteria above<br>- How patients appraise, interpret symptoms, and engage with healthcare in the diagnosis of a SPC<br>- Health-care providers' experiences, perspectives and challenges faced around the pathways to diagnosis of a SPC	- Patient and health-care provider experiences related to pathways to diagnosis for recurrent first primary cancers or metastatic cancers
**Setting**	- Primary, secondary or tertiary care in any country	-
**Study designs**	- Cohorts, case control and cross sectional studies<br>- Systematic reviews and mixed methods<br>- Qualitative studies such as interviews, focus group discussions and ethnography on the pathways to diagnosis for any SPC and associated patient and health-care provider experiences	- RCTs<br>- Studies using secondary datasets or are conference abstracts, conference proceedings and book chapters that are not focussed on the diagnostic pathways for a SPC or patient/ provider experiences of SPCs<br>- Case reports

For peer review only

Table 2: Example search strategy for MEDLINE(R)

1. exp second primary cancer/
2. second primary cancer$.ti,ab.
3. second primary malignan$.ti,ab.
4. second primary tumo$r$.ti,ab.
5. second primary neoplasm$.ti,ab.
6. or/1-5 [SPC TERMS]

7. (patient experience$ or patient know$ or patient perception$ or patient perspective$ or patient view$ or patient interpret$ or patient understand$ or patient aware$ or patient attitude).ti,ab.
8. ((health$care provider$ or health$care personnel or health$care professional$ or health$care practitioner$ or doctor$ or general practitioner$ or GP$ or consultant$) adj2 (experience$ or challenge$ or view$ or understanding$)).ti,ab.
9. or/7-8 [PATIENT & PROVIDER TERMS]

10. pathway$ to diagnos$.ti,ab.
11. diagnos$ journey.ti,ab.
12. diagnos$ pathway$.ti.
13. diagnos$ pathway$.ti,ab.
14. (diagnos$ adj2 pathway$).ti,ab.
15. route$ to diagnos$.ti,ab.
16. diagnos$ route$.ti,ab.
17. pathway$ to treatment$.ti,ab.
18. (patient appraisal or help$seeking behavio$r$ or diagnos$ interval$ or pre$treatment interval$).ti,ab.
19. (first symptom or first presentation or clinical appearance or first investigation or first referral or first specialist visit or diagnosis or treatment start).ti,ab.
20. ((patient appraisal and self$ management) or decision to consult health$care provider or health$care provider appraisal, investigations, referrals, appointment$ or planning or scheduling treatment).ti,ab.
21. or/10-20 [PATHWAYS TO DIAGNOSIS TERMS]

22. 9 or 21 [PATIENTS & PROVIDER TERMS AND PATHWAYS TO DIAGNOSIS TERMS)

23. 6 and 22 [CANCER TERMS AND PATIENTS & PROVIDER TERMS AND PATHWAYS TO DIAGNOSIS TERMS COMBINED]

24. limit 23 to humans [LIMITED TO HUMAN ONLY STUDIES]

For peer review only

Table 3: Quality Assessment tools

Tools	Study designs
CASP checklist	Systematic review<br>Case control<br>Cohorts<br>Qualitative [e.g. interviews, focus group discussions and ethnography]
McMaster Critical Review Tool & STROBE	Cross-sectional<br>Before and after
MMAT	Mixed methods
Aarhus checklist	All included studies that report and measure intervals of patient cancer journey

For peer review only

Figure 1: Pathways to treatment model

Figure 2: Model of key time points and intervals [as adapted by Weller et al, 2012]

For peer review only

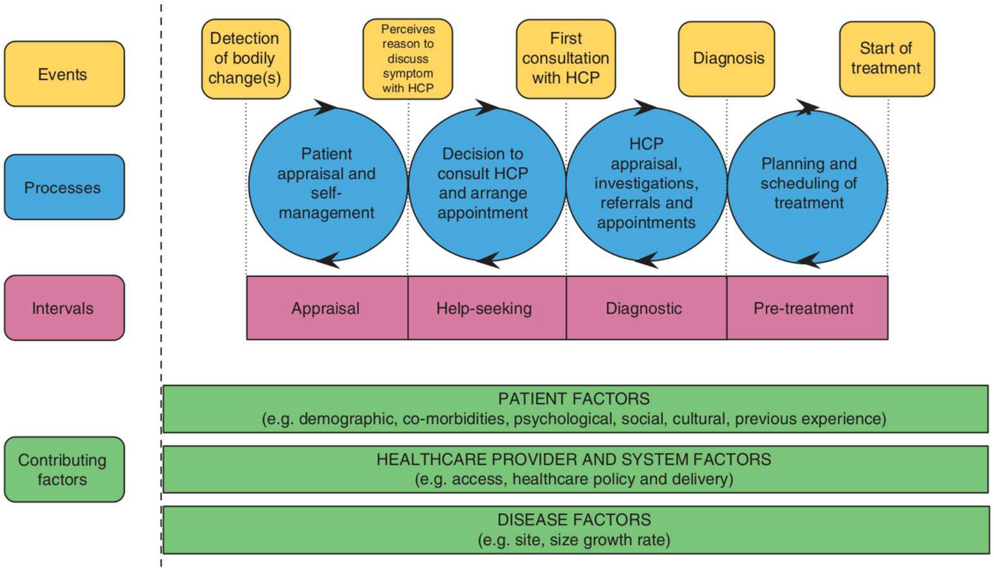

Figure 1: Pathways to treatment model

129x74mm (300 x 300 DPI)

review only

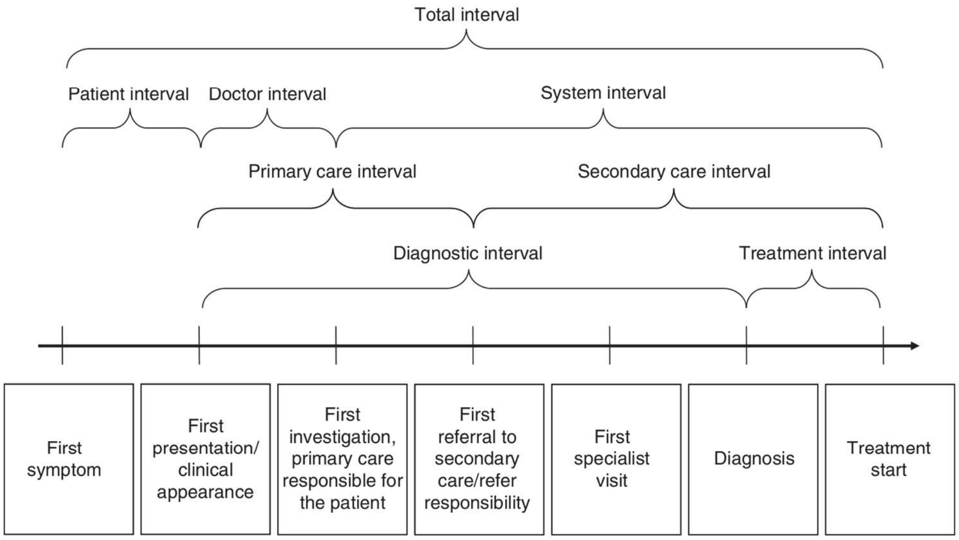

Figure 2: Model of key time points and intervals (as adapted by Weller et al, 2012)

134x72mm (300 x 300 DPI)

review only

PRISMA-P (Preferred Reporting Items for Systematic review and Meta-Analysis Protocols) 2015 checklist: recommended items to address in a systematic review protocol*

Section and topic	Item No	Checklist item	Reported on page
**ADMINISTRATIVE INFORMATION**			
Title:			
Identification	1a	Identify the report as a protocol of a systematic review	1
Update	1b	If the protocol is for an update of a previous systematic review, identify as such	N/A
Registration	2	If registered, provide the name of the registry (such as PROSPERO) and registration number	2
Authors:			
Contact	3a	Provide name, institutional affiliation, e-mail address of all protocol authors; provide physical mailing address of corresponding author	1
Contributions	3b	Describe contributions of protocol authors and identify the guarantor of the review	12
Amendments	4	If the protocol represents an amendment of a previously completed or published protocol, identify as such and list changes; otherwise, state plan for documenting important protocol amendments	N/A
Support:			
Sources	5a	Indicate sources of financial or other support for the review	12
Sponsor	5b	Provide name for the review funder and/or sponsor	12
Role of sponsor or funder	5c	Describe roles of funder(s), sponsor(s), and/or institution(s), if any, in developing the protocol	12
**INTRODUCTION**			
Rationale	6	Describe the rationale for the review in the context of what is already known	4-5
Objectives	7	Provide an explicit statement of the question(s) the review will address with reference to participants, interventions, comparators, and outcomes (PICO)	5
**METHODS**			
Eligibility criteria	8	Specify the study characteristics (such as PICO, study design, setting, time frame) and report characteristics (such as years considered, language, publication status) to be used as criteria for eligibility for the review	6-7
Information sources	9	Describe all intended information sources (such as electronic databases, contact with study authors, trial registers or other grey literature sources) with planned dates of coverage	7
Search strategy	10	Present draft of search strategy to be used for at least one electronic database, including planned limits, such that it could be repeated	19

For peer review only

Study records:			
Data management	11a	Describe the mechanism(s) that will be used to manage records and data throughout the review	8
Selection process	11b	State the process that will be used for selecting studies (such as two independent reviewers) through each phase of the review (that is, screening, eligibility and inclusion in meta-analysis)	8
Data collection process	11c	Describe planned method of extracting data from reports (such as piloting forms, done independently, in duplicate), any processes for obtaining and confirming data from investigators	8
Data items	12	List and define all variables for which data will be sought (such as PICO items, funding sources), any pre-planned data assumptions and simplifications	8-9
Outcomes and prioritization	13	List and define all outcomes for which data will be sought, including prioritization of main and additional outcomes, with rationale	8-9
Risk of bias in individual studies	14	Describe anticipated methods for assessing risk of bias of individual studies, including whether this will be done at the outcome or study level, or both; state how this information will be used in data synthesis	9
Data synthesis	15a	Describe criteria under which study data will be quantitatively synthesised	N/A
	15b	If data are appropriate for quantitative synthesis, describe planned summary measures, methods of handling data and methods of combining data from studies, including any planned exploration of consistency (such as I^2, Kendall's τ)	N/A
	15c	Describe any proposed additional analyses (such as sensitivity or subgroup analyses, meta-regression)	N/A
	15d	If quantitative synthesis is not appropriate, describe the type of summary planned	10
Meta-bias(es)	16	Specify any planned assessment of meta-bias(es) (such as publication bias across studies, selective reporting within studies)	N/A
Confidence in cumulative evidence	17	Describe how the strength of the body of evidence will be assessed (such as GRADE)	N/A

*** It is strongly recommended that this checklist be read in conjunction with the PRISMA-P Explanation and Elaboration (cite when available) for important clarification on the items. Amendments to a review protocol should be tracked and dated. The copyright for PRISMA-P (including checklist) is held by the PRISMA-P Group and is distributed under a Creative Commons Attribution Licence 4.0.**

From: Shamseer L, Moher D, Clarke M, Ghersi D, Liberati A, Petticrew M, Shekelle P, Stewart L, PRISMA-P Group. Preferred reporting items for systematic review and meta-analysis protocols (PRISMA-P) 2015: elaboration and explanation. BMJ. 2015 Jan 2;349(jan02 1):g7647.

For peer review only
